# Supplementary material for: Impact of Combined Macronutrient Diet on Amino Acids and Amines Plasma Levels
Source: Nutrients. 2025 May 16;17(10):1694. doi: 10.3390/nu17101694 (PMC12114129; doi:10.3390/nu17101694)
Supplement: Supplementary file 1 [file nutrients-17-01694-s001.zip › nutrients-3636803-supplementary.pdf]

**Supplemental Table S1**. Calibration standards.

| ANALYTE | Dynamic range of the curve (ng/ml) |
|---|---|
| Acetylcholine | 3.12-400 |
| Alanine | 173.4-88800 |
| Arginine | 679.7-87000 |
| Asparagine | 62.5-20000 |
| Aspartate | 259.37-66400 |
| Asymmetric dimethylarginine (ADMA) | 7.81-500 |
| Citrulline | 234.37-60000 |
| Creatine | 62.5-8000 |
| Gamma-aminobutyric acid (GABA) | 3.12-200 |
| Glutamate | 286.7-146800 |
| Glutamine | 15625-250000 |
| Glycine | 292.2-74800 |
| Histidine | 292.96-154800 |
| Isoleucine | 514-131600 |
| Kynurenic acid | 187.5-6000 |
| Kynurenine | 9.76-5000 |
| Leucine | 515.6-132000 |
| Lysine | 285.15-146000 |
| Methionine | 3.66-30000 |
| Phenylalanine | 1287.5-82400 |
| Proline | 224.2-114800 |
| D-Serine | 117.18-1875 |
| L-Serine | 117.18-1875 |
| Serotonin | 4.68-600 |
| Threonine | 232-118800 |
| Tryptophan | 5625-180000 |
| Tyrosine | 706.25-90400 |
| Valine | 457.8-117200 |

**Supplemental Table S2**. Multiple reaction monitoring conditions for the metabolites.

| | COMPOUND NAME | TRANSITION (M/Z) | FRAGMENTOR (V) | COLLISION ENERGY (V) | RETENTION TIME (MIN) |
|---|---|---|---|---|---|
| 1 | Acetylcholine | 146-87 | 120 | 15 | 2.15 |
| | D4-Acetylcholine | 150-91 | 120 | 15 | 2.15 |
| 2 | Alanine | 194-105 | 120 | 20 | 10.08 |
| | 13C6Bz-Alanine | 200-111 | 120 | 20 | 10.08 |
| 3 | Arginine | 279-105 | 135 | 30 | 7.68 |
| | 13C6Bz-Arginine | 285-111 | 135 | 30 | 7.68 |
| 4 | Asparagine | 237-105 | 120 | 20 | 7.46 |
| | 13C6Bz-Asparagine | 243-111 | 120 | 20 | 7.46 |
| 5 | Aspartate | 238-105 | 120 | 10 | 8.46 |
| | 13C6Bz-Aspartate | 244-111 | 120 | 10 | 8.46 |
| 6 | Asymmetric dimethylarginine (ADMA) | 307.2-105 | 70 | 20 | 8.04 |
| | 13C6Bz- Asymmetric dimethylarginine (ADMA) | 313-111 | 70 | 20 | 8.04 |
| 7 | Choline | 104-60 | 120 | 20 | 2 |
| | D4-Choline | 108-60 | 120 | 20 | 2 |
| 8 | Citrulline | 280-105 | 120 | 20 | 8.28 |
| | 13C6Bz-Citrulline | 286-111 | 120 | 20 | 8.28 |
| 9 | Creatine | 132.1-90.2 | 90 | 9 | 2 |
| 10 | Cysteine | 330-105 | 120 | 20 | 20.85 |
| | 13C6Bz-Cysteine | 342-111 | 120 | 20 | 20.85 |
| 11 | Gamma-aminobutyric acid (GABA) | 208-105 | 120 | 10 | 10.36 |
| | 13C6Bz-Gamma-aminobutyric acid (GABA) | 214-111 | 120 | 10 | 10.36 |
| 12 | Glutamate | 252-105 | 120 | 20 | 9.08 |
| | 13C6Bz-Glutamate | 258-111 | 120 | 20 | 9.08 |
| 13 | Glutamine | 251-105 | 120 | 20 | 8.17 |
| | 13C6Bz-Glutamine | 257-111 | 120 | 20 | 8.17 |
| 14 | Glycine | 180-105 | 120 | 10 | 8.79 |
| | 13C6Bz-Glycine | 186-111 | 120 | 10 | 8.79 |
| 15 | Histidine | 260-110 | 130 | 20 | 6.9 |
| | 13C6Bz-Histidine | 266-110 | 130 | 20 | 6.9 |
| 16 | Isoleucine | 236-105 | 120 | 30 | 15.9 |
| | 13C6Bz-Isoleucine | 242-111 | 120 | 30 | 15.9 |
| 17 | Kynurenic acid | 294-105 | 120 | 30 | 18.5 |
| | 13C6Bz-Kynurenic acid | 300-111 | 120 | 30 | 18.5 |
| 18 | Kynurenine | 417-122 | 120 | 10 | 19.02 |
| | 13C6Bz-Kynurenine | 429-128 | 120 | 10 | 19.02 |
| 19 | Leucine | 236-105 | 120 | 30 | 16.31 |
| | 13C6Bz-Leucine | 242-111 | 120 | 30 | 16.31 |
| 20 | Lysine | 355-188 | 120 | 20 | 14.7 |
| | 13C6Bz-Lysine | 367-194 | 120 | 20 | 14.7 |
| 21 | Methionine | 254-105 | 120 | 15 | 13.69 |
| | 13C6Bz-Methionine | 260-111 | 120 | 15 | 13.69 |
| 22 | Phenylalanine | 270-120 | 120 | 10 | 16.48 |
| | 13C6Bz-Phenylalanine | 276-120 | 120 | 10 | 16.48 |
| 23 | Proline | 220-105 | 120 | 20 | 11.03 |
| | 13C6Bz-Proline | 226-111 | 120 | 20 | 11.03 |
| 24 | D-Serine | 210-105 | 120 | 20 | 7.7 |
| | 13C6Bz-D-Serine | 216-111 | 120 | 20 | 7.7 |
| 25 | L-Serine | 210-105 | 120 | 20 | 7.99 |
| | 13C6Bz-L-Serine | 216-111 | 120 | 20 | 7.99 |
| 26 | Serotonine | 385-264 | 140 | 20 | 20.6 |
| | 13C6Bz-Serotonine | 397-270 | 140 | 20 | 20.6 |
| 27 | Threonine | 224-105 | 140 | 20 | 16.5 |
| | 13C6Bz-Threonine | 230-111 | 140 | 20 | 16.5 |
| 28 | Tryptophan | 309-159 | 120 | 10 | 16.54 |
| | 13C6Bz-Tryptophan | 315-159 | 120 | 10 | 16.54 |
| 29 | Tyrosine | 390-105 | 120 | 30 | 19.67 |
| | 13C6Bz-Tyrosine | 402-111 | 120 | 30 | 19.67 |
| 30 | Valine | 222-105 | 120 | 30 | 13.61 |
| | 13C6Bz-Valine | 228-111 | 120 | 30 | 13.61 |
